# Acute Metabolic Response in Adults to Toddler Milk Formulas with Alternating Higher and Lower Protein and Fat Contents, a Randomized Cross-Over Trial

**DOI:** 10.3390/nu13093022

**Published:** 2021-08-29

**Authors:** Emily Newton-Tanzer, Hans Demmelmair, Jeannie Horak, Lesca Holdt, Berthold Koletzko, Veit Grote

**Affiliations:** 1 Division of Metabolic and Nutritional Medicine, Dr. von Hauner Children’s Hospital, LMU University Hospital Munich, 80337 Munich, Germany; emily.newtontanzer@med.uni-muenchen.de (E.N.-T.); hans.demmelmair@med.uni-muenchen.de (H.D.); Jeannie.horak@med.uni-muenchen.de (J.H.); veit.grote@med.uni-muenchen.de (V.G.); 2 Institute of Laboratory Medicine, LMU University Hospital Munich, 81377 Munich, Germany; lesca.holdt@med.uni-muenchen.de

**Keywords:** toddler milk, milk protein, diet, branched-chain amino acids, insulin, glucose, triglycerides, urea, postprandial phase, the early protein hypothesis

## Abstract

Protein intake in early life influences metabolism, weight gain, and later obesity risk. As such, a better understanding of the effects of protein intake on the postprandial metabolism and its dynamics over time may elucidate underlying mechanisms. In a randomized crossover study, we observed fasted adults who consumed two isocaloric toddler milk formulas concentrated as meals of 480 kcal with 67 g of carbohydrates 30 g (HP) or 7 g (LP) protein, and 10 g or 20 g fat, respectively. Anthropometry and body plethysmography were assessed, and blood samples collected at baseline and over five hours. Time-specific concentrations, areas under concentration curves (AUC), and maximum values of metabolites were compared by paired t-tests to examine the effects of protein content of toddler milks on postprandial plasma concentrations of insulin, glucose, branched-chain amino acids (BCAA), urea and triglycerides. Twenty-seven men and women aged 26.7 ± 5.0 years (BMI: 22.2 ± 2.5 kg/m^2^) (mean ± SD) participated. BCAA AUC, and Cmax values were significantly higher with HP than LP (144,765 ± 21,221 vs. 97,089 ± 14,650 µmol·min/L, *p* < 0.001; 656 ± 120 vs. 407 ± 66 µmol/L, *p* < 0.001), as were insulin AUC and Cmax values (6674 ± 3013 vs. 5600 ± 2423 µmol·min/L, *p* = 0.005; 71 ± 37 vs. 55 ± 28 µmol/L, *p* = 0.001). Higher glucose, urea, and triglyceride concentrations occurred in the late postprandial phase (≥180 min) with HP. In conclusion, we noted that higher milk protein intake induces increased postprandial BCAA concentrations for at least 5 h and led to higher initial insulin secretion. Gluconeogenesis due to an influx of amino acids and their degradation after HP meal might explain the late effects of protein intake on glucose and insulin.

## 1. Introduction 

The early protein hypothesis suggests that a higher protein supply fed to infants with conventional infant formula, as compared with breastmilk, induces an increased early weight gain and an increased risk of obesity in later life [1,2,3,4]. A large double-masked, randomized trial, the European Childhood Obesity Project (CHOP), provided evidence that higher milk protein intake in the first year of life markedly increased body mass index (BMI), body fatness, and the prevalence of obesity at ages 2 and 6 years [5,6,7]. Due to the lower supply of protein in human milk as compared to cow’s milk and conventional formula, infants fed conventional formula receive higher quantities of protein than breast-fed infants [8]. Milk protein, predominantly composed of casein and whey, is high in branched-chain amino acids (BCAAs), which can induce an increased secretion of insulin and insulin-like-growth factor I (IGF-1) [9]. Together with BCAAs, insulin and IGF-1 activate the mammalian target of rapamycin signaling pathway, particularly the mammalian target of the rapamycin complex 1 (mTORC1), critical for adipogenesis and the maintenance of fat tissue [9,10].

Observational studies have suggested that the effect of protein intake on weight and BMI is not restricted to infancy but can also be seen during toddlerhood—although the available evidence is limited [11]. The ongoing Toddler Milk Intervention Study (TOMI) (clinicaltrial.gov: NCT02907502, accessed on 30 August 2021) was initiated to study the effects of two isocaloric toddler milk formulas with higher and lower concentrations of protein, with an adaptation of fat content to maintain equal caloric density, consumed by young children between 12 and 24 months of age. As sequential blood sampling is not feasible in young children, the TOMI study cannot provide insight into the short-term postprandial effects of the differing milks that could contribute to the effects of protein intake on growth. 

Therefore, in the present study, we attempt to explore potential mechanisms by analyzing the acute effects of the two TOMI milk formulas as test meals on postprandial metabolism in healthy, young adults. We hypothesize that the consumption of a higher protein test meal will be followed by larger postprandial increases in plasma concentrations of insulin and BCAA, as compared to the lower protein formula. 

## 2. Materials and Methods 

### 2.1. Subjects 

**Figure 1:**
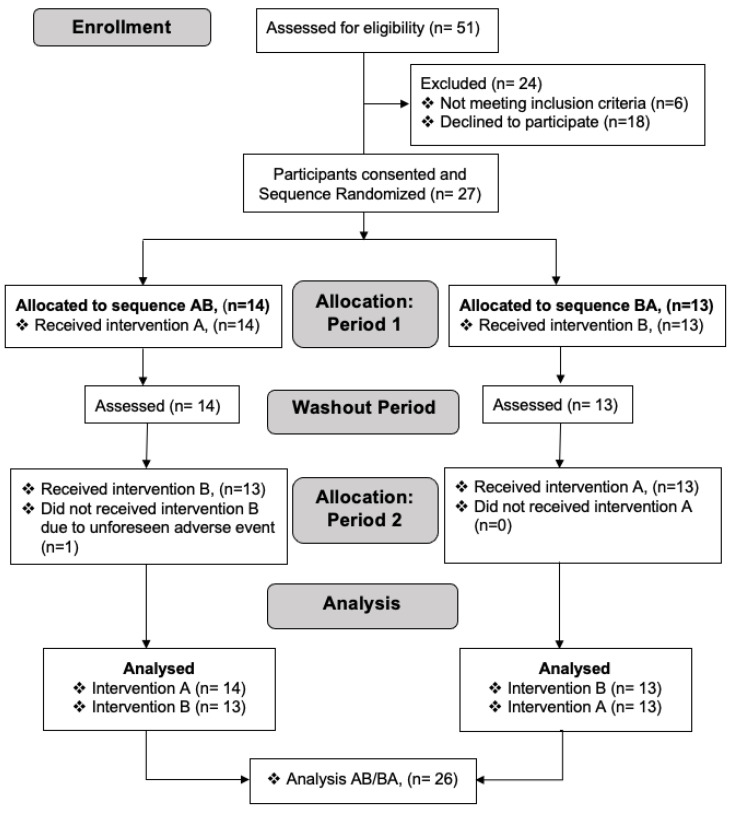
A CONSORT (Consolidated Standards of Reporting Trials) diagram depicting the process of recruitment, selection, randomization and analysis of participants. Due to an unforeseen adverse event (lactose intolerance), one participant (*n* = 1) did not perform intervention B during period 2 of allocation and thus was excluded in the analysis of intervention B.

Participants were invited for the study from May to September 2019 at the Dr. von Hauner Children’s Hospital, LMU University Hospital, Munich, Germany. A CONSORT (Consolidated Standards of Reporting Trials) diagram of the progress from recruitment through to the completion of the study is included in the Figure 1. Healthy, unmedicated volunteers could participate in the study if they were between 18 to 40 years old, had a BMI between 18 and 25 kg/m^2^, and had no known metabolic, cardiovascular, or other disorders, including lactose intolerance. Upon pre-screening, two appointments were made for each intervention, separated by 5–10 days (with a mean of 6.7 days). Of 51 screen subjects, we could include 27 subjects (Figure 1). 

Prior to the first intervention, the participants completed an informed consent form, as well as a health and well-being questionnaire. Data on fitness and activity level, country of origin, education, and occupation were collected through questionnaires. Upon completion, anthropometric measurements were performed, including height, weight, and waist circumference. BMI was then calculated, and body composition was determined using air displacement plethysmography (BOD POD©, COSMED, Fridolfing, Germany) to assess body fat content and percentage of body fat. A venous, indwelling catheter (Safety-Multifly^®^ Sarstedt) was inserted into the participants’ forearm to collect blood samples. 

### 2.2. Test Meals and Interventions

The participants consumed two isocaloric toddler milk formulas with alternating higher and lower protein, and fat content as a standardized test meal. Both formulas were originally designed for the ‘Toddler Milk Intervention Study- TOMI’ (clinicaltrial.gov: NCT02907502, accessed on 30 August 2021), produced and provided by Nestec, Ltd., Vevey, Switzerland, now renamed to Société des Produits Nestlé S.A., Vevey, Switzerland. In order to maintain double-blinding, the milk formula was pre-packaged by the manufacturer in individual-dosed cans labeled with A or B. Each milk powder was dissolved in 500 mL of water at a temperature of 40 °C prior to consumption. The utilized quantity of milk formula in the present study was chosen to be approximately equal to the caloric content of a single adult meal and corresponds to double the concentration used for the preparation of the milk formula for children participating in the TOMI study. Thus, the higher protein, lower fat (HP) test meal contained 2010 KJ/480 kcal energy, 30 g protein, 67 g carbohydrates and 10 g fat per 500 mL. The lower protein, higher fat (LP) test meal contained 2010 KJ/480 kcal energy, 7 g protein, 67 g carbohydrates and 20 g fat per 500 mL. 

A randomization list and the transfer of the planned assignments to sealed and consecutively numbered envelopes was prepared by a person not involved in the conduct and lab analysis of the study. The list was produced using a randomly permuted sequence of A and B with a block length of 2 or 4 and a consecutive ID number. Group A was allocated the product sequence AB and the group B the product sequence BA. Product allocation after informed consent was performed by opening the respective sealed envelope with the id containing the product code for the first and second appointment. At each appointment, participants arrived after an overnight fast of at least 12 h to the study site. Fasted blood samples were taken (time = 0 min), followed by anthropometric measurements, which were only performed during the first appointment. The pre-packaged milk formulas A and B were chosen for each trial appointment, according to the allocation. After insertion of the venous catheter the formulas were consumed within a 5-min time period, while sitting at a table. Initially, only 450 mL of water was mixed with the test milk formula. Upon consumption of the 450 mL, an additional 50 mL of water was mixed with the leftover milk at 40 °C, in order to ensure that all formula was consumed. Trial periods for all subjects began at 8:00 a.m., and the time of meal intake for all subjects did not differ more than one hour.

Timing for blood samples began once the full test meal was consumed, and venous blood samples were taken at 15, 30, 60, 90, 120, 180, 240, and 300 min postprandial. With the exception of the first samples of the first subject, all blood samples were taken within a minute of each time interval. At each of the time points, including the basal fasted blood samples, serum and EDTA blood samples were taken (using S-Monovette^®^, Sarstedt) to measure glucose, insulin, BCAA, triglycerides, and urea. In the fasted blood samples additionally full blood count, CRP, ALAT, and ASAT were determined to evaluate the overall health status of the subjects. The same arm was used for the venous catheter at each of the two interventions.

During the test period, subjects were asked for any complaints or adverse events. Throughout the entire trial, subjects only carried out simple tasks and refrained from any significant physical activity, with no excessive or strenuous physical activity. The subjects did not drink or eat anything during the first 120 min of the sample collection period, after which they were allowed to drink water. All test subjects requested to drink water after 120 min of the observation period.

The procedure for the second trial was identical to the first, except that anthropometric measurements were not taken, and the subjects consumed the second milk formula (either A or B, respectively). The subjects were encouraged not to alter their normal routines between study tests and were asked to report any major change of their lifestyle (diet, physical activity, or alcohol consumption) between the tests, though none were reported. 

### 2.3. Laboratory Measurements

Alanine Aminotransferase (ALTPM: ACN 8681, Roche/Hiatchi cobas c 701/702), Aspartate Aminotransferase (ASTPM: ACN 8680, Roche/Hiatchi cobas c 701/702), C-Reactive Protein (AU 5800, Beckman Coulter, Brea, CA, USA), and a full blood count (Fluorescence, XE 5000 or XN 9000, Sysmex) and flow cytometry (Coulter LH 750, Beckman Coulter) were determined from the fasted serum and EDTA samples, respectively, and glucose (enzymatic UV assay, AU 5800, Beckman Coulter), insulin (Elektro Chemical Luminescence Immuno Assay, Cobas 8000e702, ROCHE Diagnostics, Basel, Switzerland), urea (Kinetic UV assay, AU 5800, Beckman Coulter), and triglycerides (enzymatic colour assay, Cobas 8000 c701, ROCHE) for all collected time points by the Institute of Laboratory Medicine at the University Hospital, LMU Munich. For BCAA (valine, leucine and isoleucine) determination EDTA samples were centrifuged (10 min, 1500× *g*, 4 °C) and plasma was stored at −80 °C. After collection of all the samples, plasma was analyzed via HPLC-ESI-MS/MS on an API-2000 Triple-Quadrupole-MS instrument from Sciex (Concord, ON, Canada), as previously described in Harder et al. with slight modifications to make the method more robust and reproducible [12]. For sample preparation, 50 µL plasma samples were directly pipetted and mixed into the methanolic internal standard solution in order to obtain a fluffy homogenous protein precipitate. All other steps were performed according to Harder et.al. In addition, the calibration curve ranged from 10 µM to 1000 µM (10, 25, 50, 100, 200, 500, 750 and 1000 µM) with double calibrant injections at 50 and 200 µM to stabilize the calibration curve. For each wellplate batch analysis, two calibration curves were analyzed at the start and at the end of sample analysis. Note that only calibration curves with a correlation coefficient of at least 0.9990 were employed for quantification using the MultiQuant 3.0 software from Sciex. In addition, six equidistant quality control (QC) samples (pool of all samples) and 2 × 2 amino acid control plasma samples (CP1 and CP2 from Recipe) were co-analyzed with the plasma samples to ensure highest possible accuracy and precision of all quantitative results.

### 2.4. Sample Size, Statistics and Data Analysis 

Area Under the Time Concentration Curves (AUC) were using trapezoidal functions for insulin, which was the primary outcome measure, as well as secondary outcomes measures including postprandial glucose, triglycerides, urea, and BCAAs. Total BCAA was calculated as the sum of valine, leucine, and isoleucine. Further exploratory measures, such as fasted baseline concentrations, concentration maximums (Cmax), and time of maximum concentration (Tmax) were also calculated and assessed.

Based on studies by Hirsch et al. [13] and Nilsson et al. [14], we assumed a standard deviation (SD) of 5.1 µmol·min/L in insulin AUC and a plausible difference of the AUCs between HP and LP milk formulas of 3.7 µmol·min/L. Using an error level of 5% and a statistical power of 80% in a one-sided test, a required sample size of 25 subjects was determined. 

Comparisons between the primary and secondary outcomes, as well as exploratory outcome measures between the two milk formulas were performed with paired t-tests or Wilcoxon rank test when considered appropriate. For additional explorative analysis, linear regression with cluster options (by subject) was used to test for the effects of other factors, such as BMI, gender and age on glucose, insulin, triglycerides, urea and BCAA. Statistical significance was assumed at maximum error probability of 0.05. All statistical analyses were performed with the software SPSS v26 (IBM, Armonk, NY, USA), and Stata 15.1 (StataCorp, College Station, TX, USA). 

## 3. Results

### 3.1. Participants 

Twenty-seven subjects (15 females, 56%, 12 males, 44%) participated, as shown in Table 1. 

One subject dropped out after the first test meal (higher protein meal) due to reported symptoms of food intolerance. The subject denied having been aware of any lactose or food intolerance. Thus, 26 subjects were available for the main outcome analysis and 27 and 26 subjects for any HP and LP, respectively, summary statistics. No other adverse effects occurred during the entire trial. CRP and liver enzymes were all within reference ranges, and all subjects were deemed healthy. All participants had or were undergoing an education at university level, with the majority (81%) being students. Most participants (81%) were of European descent, while 3 participants were of Asian descent (11%), and 1 participant each was of African and Indian descent, respectively.

Body plethysmography showed a body fat percentage of 25.4 ± 1.4% (mean ± SD) in females and 15.7 ± 1.4% in males. Most participants reported to be physically active 2–3 times per week (38.5%), followed by participants who exercised more than 6 times per week (30.8%), 4–5 times per week (19.2%), and less than once per week (11.5%). 

### 3.2. Blood Glucose, Insulin, Urea, and Triglyceride Responses

**Figure 2:**
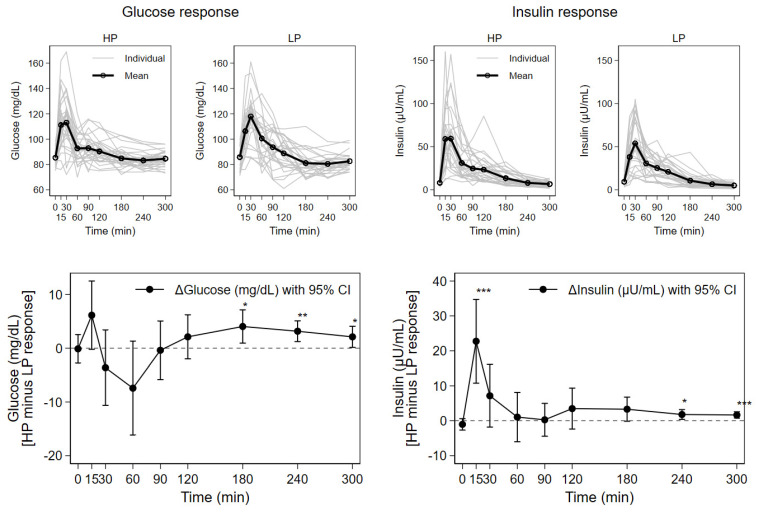
Response of serum concentrations of glucose and insulin after consumption of higher (HP) and lower protein (LP) test meals over time for 27 subjects; mean with individual concentration curves by test meal as well as mean individual difference (HP minus LP) with 95% CI (* *p* < 0.05, ** *p* < 0.01, *** *p* < 0.001).

Fasted baseline concentrations, Cmax, Tmax, as well as AUC values for glucose, insulin, urea, and triglycerides are shown in Table 2. Mean and individual concentration curves for glucose and insulin responses over time are displayed in Figure 2, together with the mean difference between the HP and LP test meals for individual metabolic responses with a 95% confidence interval (CI). A comparison of baseline, fasted values revealed no significant differences prior to consumption of the study milks. 

The postprandial curves for glucose and insulin show initial steep increases, with peak concentrations reached until approximately 30 min. Subsequently, the concentration curves sharply declined until 60 min, and then more gradually for the final three hours of the observation period. In comparison to the LP meal, glucose was somewhat higher directly after HP meals, then lower and again higher from 120 min onwards, while insulin peaked early and was always higher after HP meals. Concentration maximums and AUC for insulin were significantly higher for the HP compared to the LP milk meals (Table 2). These concentrations showed approximately 18% higher AUC values, and a 30% higher concentration maximum for the HP compared to the LP milk meals. Significant differences between mean postprandial insulin concentrations for the HP compared to the LP milk were seen at 15 min and the two last observation time points (Figure 2): at 15 min (61 ± 34 vs. 38 ± 20 µU/mL, *p* < 0.001), 240 min (8 ± 4 vs. 6 ± 4 µU/mL, *p* = 0.015) and 300 min (7 ± 3 vs. 5 ± 3 µU/mL, *p* < 0.001), respectively.

The time points of concentration maximums (Tmax) for insulin and glucose varied widely between subjects, but without significant influence by the test meals. The maximum glucose concentrations after both test milks were similar, and there were no significant differences in AUC values for glucose. However, significantly higher mean glucose concentrations after HP versus the LP test meals were observed at 180 min (85 ± 7 vs. 81 ± 10 mg/dL, *p* < 0.05), 240 min (83 ± 6 vs. 80 ± 7 mg/dL, *p* = 0.003), and 300 min (85 ± 6 vs. 83 ± 6 mg/dL, *p* = 0.036).

The concentration curves for urea and triglycerides showed gradual changes over the trial period for both the HP and the LP milk formulas. For urea, mean postprandial concentrations increased steadily until the end of the study (300 min) for HP, while the mean postprandial concentrations after consumption of LP showed a gradual decreasing trend. Significantly higher values after HP than LP were observed for the last two measurements at 240 min (27 ± 8 vs. 23 ± 7 mg/dL, *p* = 0.001) and 300 min (27 ± 7 vs. 23 ± 7 mg/dL, *p* = 0.001). The mean concentration curves for triglycerides for both HP and LP milk meals were characterized by similar, gradual developments until the end of the observation period with a significant difference in mean concentrations observed at 300 min (106 ± 48 vs. 92 ± 37 mg/dL, *p* = 0.041).

### 3.3. Postprandial Plasma Branched-Chain Amino Acids

**Figure 3:**
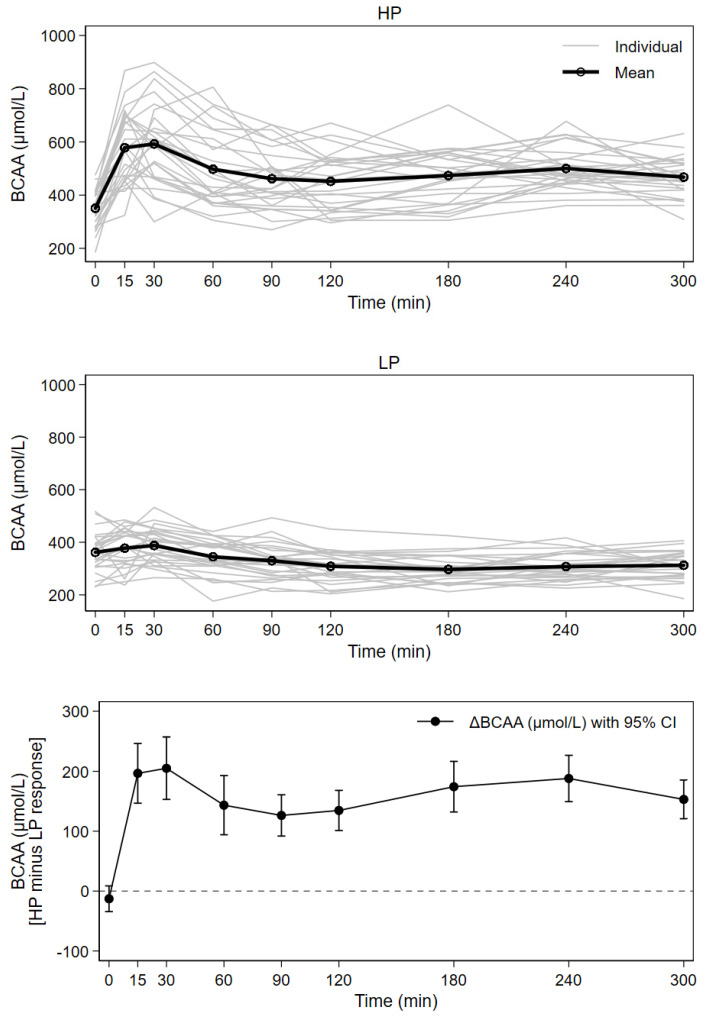
Response of serum concentrations of BCAA after consumption of higher (HP) and lower protein (LP) test meals over time for 27 subjects; mean with individual concentration curves by test meal as well as mean individual difference (HP minus LP) with 95% CI (all time points except time = 0, *p* < 0.001).

Fasted baseline concentrations, Cmax, Tmax, and AUC values for BCAAs are shown in Table 3. BCAA concentrations over time by test meal and the difference between test milk meals is depicted in Figure 3.

Both plasma BCAA AUCs and their concentration maximums were significantly higher after the intake of HP compared to LP; HP curves were characterized by steep initial increases directly after milk consumption. Significantly greater BCAA concentrations at all postprandial points were observed after HP. Approximately 37%, 83% and 87% higher concentration maximums were observed for the BCAAs valine, leucine and isoleucine, respectively. BCAAs showed a 49% higher AUC and 61% higher concentration maximums after HP compared to LP.

The time between milk intake and Cmax was highly variable (Table 3). In the HP group, approximately one third of all participants did not reach concentration maximums until at least two hours postprandially. In the LP group, all subjects reached a concentration maximum within 60 min, though the majority of participants (24 subjects) reached BCAA concentration maximums by 30 min.

Tmax in the HP group was reached significantly later for BCAAs than for insulin (*p*= 0.001), and there was no association of BCAAs with glucose or insulin maximum concentrations. Maximum concentrations and AUCs of BCAAs were not associated with maximum concentrations or AUC values of insulin.

### 3.4. Effects of Age, Gender and Anthropometry on Metabolic Response

Age, gender, and anthropometrics (weight, BMI and fat mass) of participants were examined in relation to the AUCs of insulin, glucose, BCAAs and urea. BMI, weight, and age, but not fat mass correlated negatively and significantly with AUC insulin values (r_BMI_ = −0.47, r_weight_ = −0.44, r_age_ = −0.36; all *p* ≤ 0.009). In a linear regression model, including BMI, age, gender and the study product (HP vs. LP), 1 kg/m^2^ in BMI reduced the AUC insulin value by 472.5 µmol·min/L—corresponding to a 7–8% change in the AUC, and 1 year of age reduced the AUC insulin by 191.5 µmol·min/L.

There were no other significant differences, except for gender and urea, where men displayed significantly higher concentrations throughout the entire study for both test meals.

## 4. Discussion

Our results clearly show the marked impact of milk protein intake on postprandial insulin and glucose serum concentrations. The consumption of HP led to increases in insulin secretion, initially accelerating glucose clearance, with a consecutive decrease in insulin concentrations until the end of the study. As the carbohydrate load for formulas was identical, the preliminary rise in insulin can be attributed to the insulinotropic aspects of milk proteins [15,16]. However, towards the end of the trial, insulin, glucose, and urea showed significant concentration differences after HP versus LP. This appears to reflect a dual-metabolic mechanism, intrinsically linked to the observed BCAA concentrations that remained elevated after HP for the entirety of the trial. The excess supply of postprandial BCAAs, and potentially other amino acids, could thus contribute to increased weight gain in infants fed a higher protein formula [5,6]. This can strongly influence the risk for negative health outcomes, as observational studies have convincingly shown that early weight gain is associated with higher body mass index (BMI) and other indicators of obesity in later life [17,18].

Pediatric studies examining the role of higher milk protein intake on infants and children have predominantly focused on growth effects and in part to other long-term outcomes such as obesity risk. In the Childhood Obesity Project (CHOP), infants were randomly assigned formulas with higher and lower protein content that were consumed until twelve months of life. Fasted blood samples taken at six months indicated that infants who consumed the higher protein formula had decreased serum glucose, and increased concentrations of BCAAs, while even lower BCAA concentrations were observed in a breastfed reference group [19,20]. These results support the early protein hypothesis, suggesting that increased protein supply in early life elevates concentrations of insulinogenic amino acids, and thereby increases growth mediator release, such as insulin and IGF-1 [5,21]. Other pediatric studies similarly determined that infants fed higher protein formula had significantly higher concentrations of plasma amino acids and serum urea than breast-fed infants [22,23,24], while infants fed lower protein showed serum urea and plasma amino acids concentrations similar to breastfed infants [25,26].

Although studies in adults have focused on protein intake in the acute postprandial phase, there are consistencies in the data that support our hypotheses. Panahi et al. showed that postprandial glycemia and insulinemia are closely linked to meal composition, presenting lower insulin responses per kcal fat than protein [27]. We observed similar results, with significant differences in Cmax for insulin after HP than LP that an adaptation of higher and lower fat content to maintain equal caloric density. Our results were further consistent with those of Nilsson et al. [28], as we observed significant differences in AUCs and Cmax for BCAAs after HP compared to LP. Most recently, Shahkhalili et al. observed the effects of three different infant formula meals with varying types and amounts of milk protein and similar carbohydrate and fat contents on 29 adults. Their results showed that the increased intake of partially hydrolyzed whey resulted in an increase of insulin concentrations, as well as a higher glucose response [29]. To our knowledge, this is the only other study that compared the effects of infant milk formulas in adults, though their sampling stopped at 180 min postprandial, and it remains unclear whether the greater insulin response by the higher protein load applies only to hydrolyzed whey protein or also applies for intact protein.

Milk protein composition, primarily composed of whey and casein, is particularly relevant regarding metabolic responses. Whey protein is rapidly digested, triggering specific gastric incretins, such as glucagon-like peptide (GLP-1) and glucose-dependent insulinotropic polypeptide (GIP) [15,30]. Whey is also high in leucine, assumed to be a crucial dietary component in adipogenesis due to mTORC1 signaling [28,31,32,33,34], while casein has been described to enhance IGF-1 serum levels [14,35]. In our present study, we did not analyze IGF-1 levels, as previous data suggested that the acute postprandial effects of IGF-1 are minimal compared to those of insulin [36,37].

We noted significant differences in mean concentration of insulin, glucose and urea after HP compared to LP after 240 min. Krezowski et al. [38] showed that a protein-stimulated insulin response in healthy adults did not necessarily parallel the rise in amino acids, suggesting other hormonal or metabolic mechanisms in the insulin release observed after two hours postprandial. As we also found no association of Cmax or AUCs of BCAAs with those of glucose or insulin, and Tmax for BCAAs was significantly later than for insulin, amino acids appear to have continuously cumulated after the initial insulin peak. We thus postulate that an increased supply of BCAAs, and potentially other amino acids, leads to increased amino acid degradation, triggering glucogenesis, and subsequently insulin stimulation. These results were further substantiated by significant differences in mean urea concentrations between HP and LP also observed at 240 and 300 min, suggesting an increase in degradation products as a result of increased amino acid breakdown [39]. However, as of the BCAAs, only valine is purely glucogenic, we speculate whether interplay from other glucogenic amino acids took place, which requires further examination.

It is unclear to which extent these acute metabolic effects observed in young, healthy adults can be extrapolated to children. While multiple observational studies have shown that early protein intake is associated with higher BMI in early childhood reflecting assumed early metabolic programming of adiposity [2,40,41,42,43], other research in juveniles suggests that higher protein intake in pre-teen and teen years may increase lean mass and is thereby associated with pubertal growth rather than adiposity programming [44,45]. We observed that lower body mass in adults resulted in decreased postprandial insulin. We also noted a negative correlation between insulin and increasing age.

### Strengths and Limitations

The limitations of our study include lack of a control for gastric emptying, and no measure of other endocrine factors that may have interacted with or influenced the pre- and postprandial phases. Further, we focused on the interplay of BCAAs in the postprandial metabolism, while other amino acids, particularly glucogenic, may have also played a role. Although our study included a modest sample size, we found clear group differences despite intra- and inter-individual variability in blood metabolite concentrations.

The strengths of our study include the relatively long observation period of five-hours, the double-blind randomized design, and strictly controlled study conditions. Participants consumed a standard milk meal and completed no major physical activity for the duration of the observation period; therefore, we can exclude the confounding effects of exercise on the postprandial response. In an attempt to avoid any alterations in blood values, the venous catheter was always inserted into the same arm and anthropometric measurements were standardized based on the previously enacted TOMI study protocol.

## 5. Conclusions

In conclusion, our results show that a high milk protein supply affects acute postprandial BCAA and insulin concentrations in healthy adults. The initial rise in insulin is likely due to the ingestion of glucose combined with BCAAs, while effects observed after two-hours are likely related to amino acid degradation and subsequent gluconeogenesis, due to the cumulative increase in circulating BCAAs supply over time. Though these outcomes cannot be directly compared to postprandial metabolic effects in young children, they can contribute to better understanding the mechanisms behind high milk protein supply on metabolism and growth in early life.

## Figures and Tables

**Table 1:** Baseline characteristics of study subjects (*n* = 27).

Baseline Variable	Mean ± SD
Age (years)	26.4 ± 5.0
Weight (kg)	68.3 ± 12.4
Height (cm)	173.9 ± 10.2
BMI (kg/m^2^)	22.2 ± 2.5
Waist circumference (cm)	80.7 ± 7.0

**Table 2:** Fasted baseline concentrations (mg/dL; µU/mL), maximum concentration (Cmax), mean time at maximum concentration (Tmax; minutes) and AUC values (mean ± SD; µmol·min/L) for glucose, urea, triglycerides and insulin after intake of higher and lower protein test milks for the 27 subjects.

Metabolite	Higher Protein	Lower Protein	*p*-Value *
Glucose (mg/dL)			
Baseline	86 ± 6	86 ± 9	0.929
Cmax	119 ± 19	119 ± 18	0.866
Tmax	37 ± 29	45 ± 38	0.251
AUC	27,301 ± 1868	26,826 ± 2165	0.132
Insulin (µU/mL)			
Baseline	8 ± 4	9 ± 5	0.202
Cmax	71 ± 37	55 ± 28	<0.001
Tmax	24 ± 11	27 ± 10	0.247
AUC	6674 ± 3013	5600 ± 2423	0.005
Urea (mg/dL)			
Baseline	25 ± 9	27 ± 8	0.287
Cmax	28 ± 8	27 ± 8	0.512
Tmax	235 ± 90	23 ± 27	<0.001
AUC	7873 ± 2459	7259 ± 2254	0.110
Triglycerides (mg/dL)			
Baseline	85 ± 46	80 ± 49	0.458
Cmax	110 ± 50	110 ± 53	0.961
Tmax	246 ± 96	201 ± 111	0.132
AUC	27,945 ± 14338	26,619 ± 12,862	0.455

* *p* value from paired t test and Wilcoxon rank test for Tmax.

**Table 3:** Fasted baseline concentrations (µmol/L), maximum concentration (Cmax), mean time at maximum concentration (Tmax; minutes) and AUC values (mean ± SD; µmol·min/L) for total BCAAs, valine, leucine, and isoleucine for the higher protein and lower protein study milks for 27 subjects.

Amino Acid	Higher Protein	Lower Protein	*p*-Value *
Total BCAA			
Baseline	349 ± 71	362 ± 74	0.229
Cmax	656 ± 120	407 ± 66	<0.001
Tmax	101 ± 106	34 ± 57	0.024
AUC	144,765 ± 21,221	97,089 ± 14,650	<0.001
Valine			
Baseline	174 ± 37	183 ± 38	0.204
Cmax	284 ± 63	207 ± 39	<0.001
Tmax	118 ± 112	43 ± 60	0.009
AUC	67,465 ± 11,871	50,385 ± 7944	<0.001
Leucine			
Baseline	114 ± 26	115 ± 25	0.828
Cmax	242 ± 40	133 ± 21	<0.001
Tmax	77 ± 96	35 ± 59	0.136
AUC	49,611 ± 7220	30,419 ± 4970	<0.001
Isoleucine			
Baseline	61 ± 15	64 ± 21	0.391
Cmax	141 ± 30	75 ± 18	<0.001
Tmax	64 ± 87	35 ± 59	0.277
AUC	27,689 ± 4632	16,285 ± 3314	<0.001

* *p* value from paired t test and Wilcoxon rank test for Tmax.

## Data Availability

Data described in the manuscript, code book and analytic code will be made available upon request pending application and approval.

## Abbreviations

HP: higher protein formula; LP, lower protein formula; BMI, body mass index; SD, standard deviation; AUC, area under the concentration curve; BCAA, branched-chain amino acids; Cmax, concentration maximum; Tmax, time of maximum concentration; CI, confidence interval.
